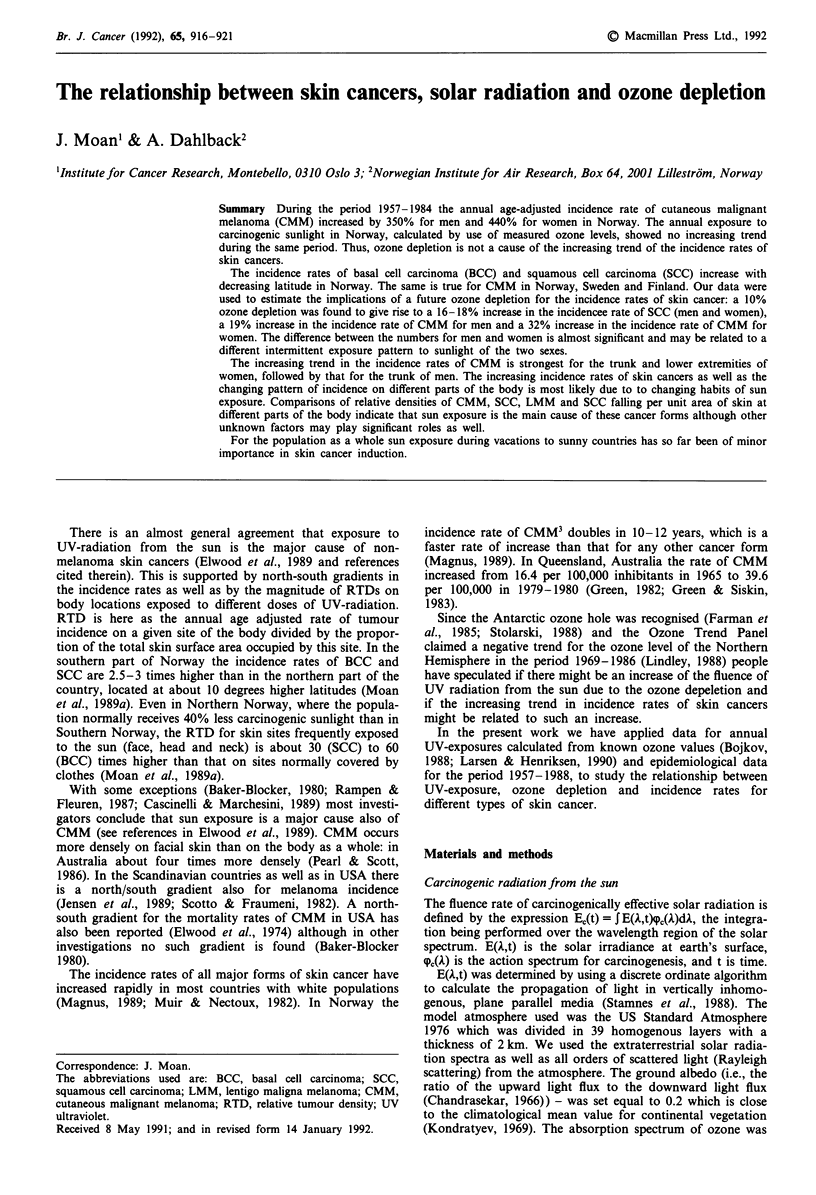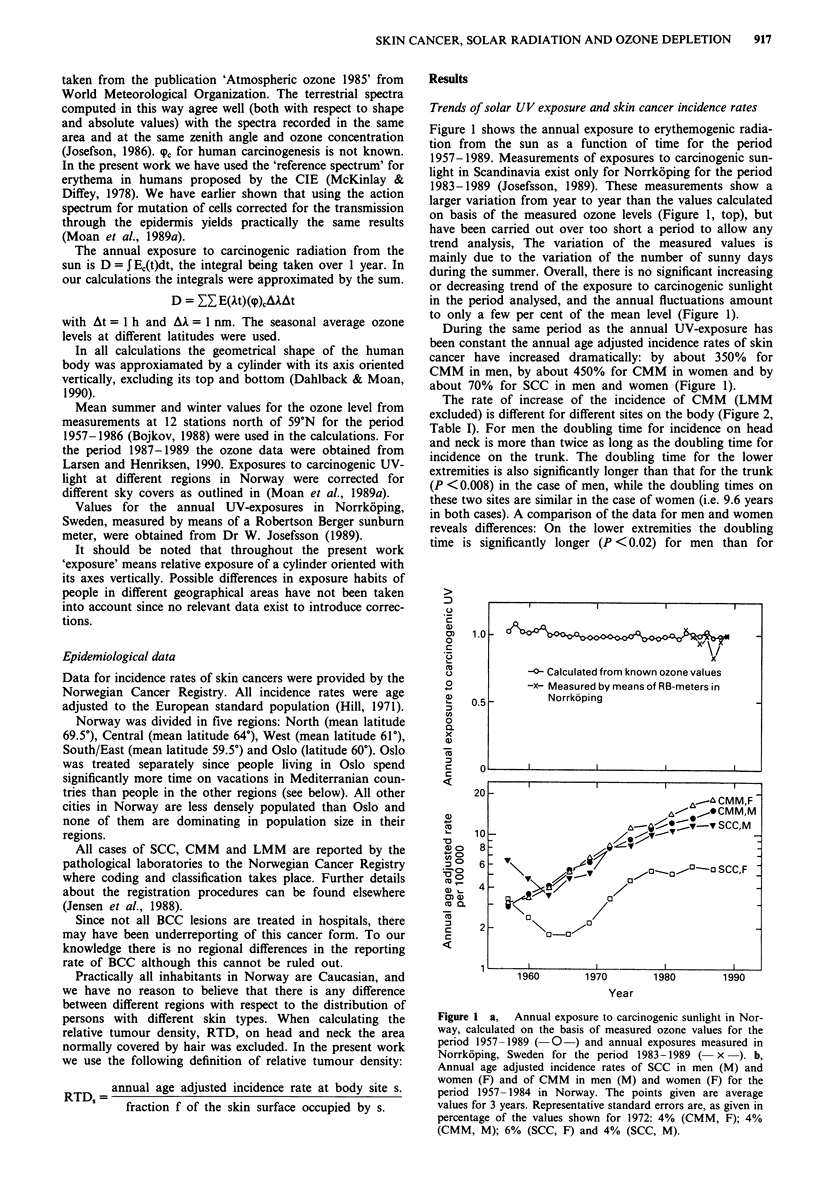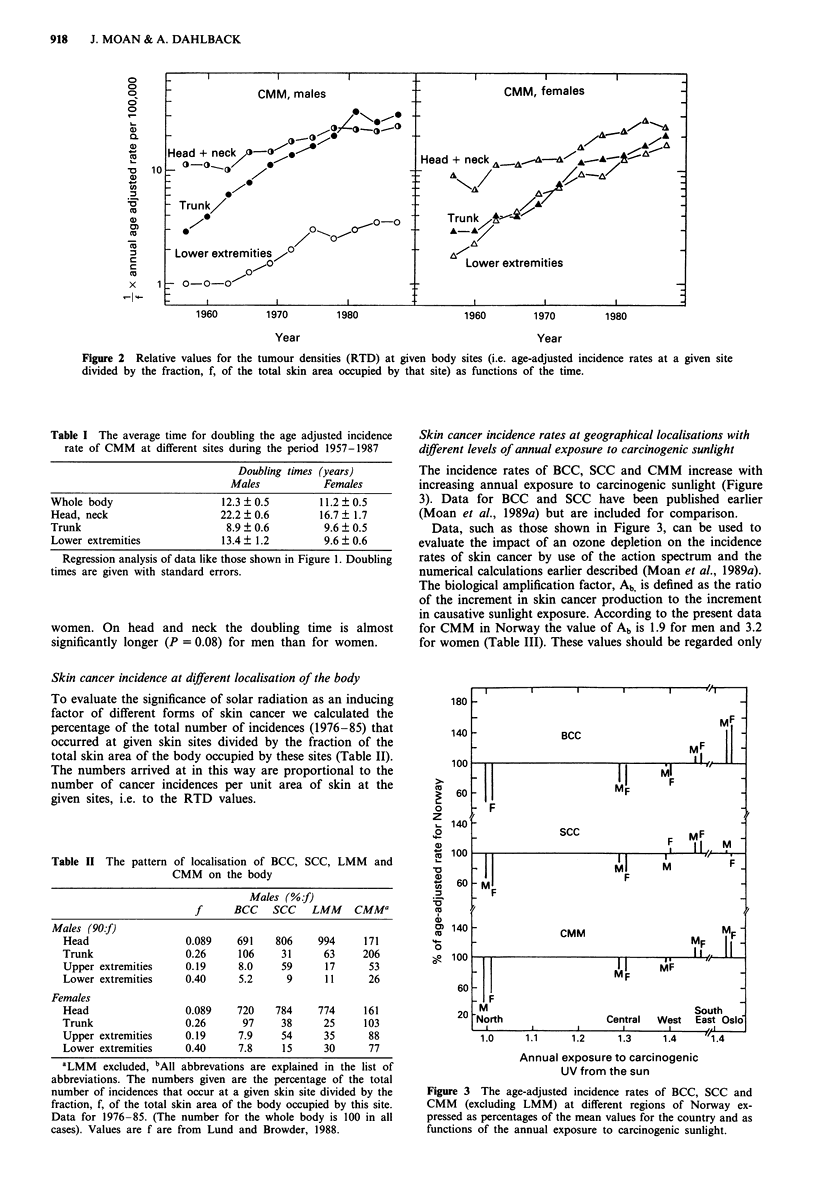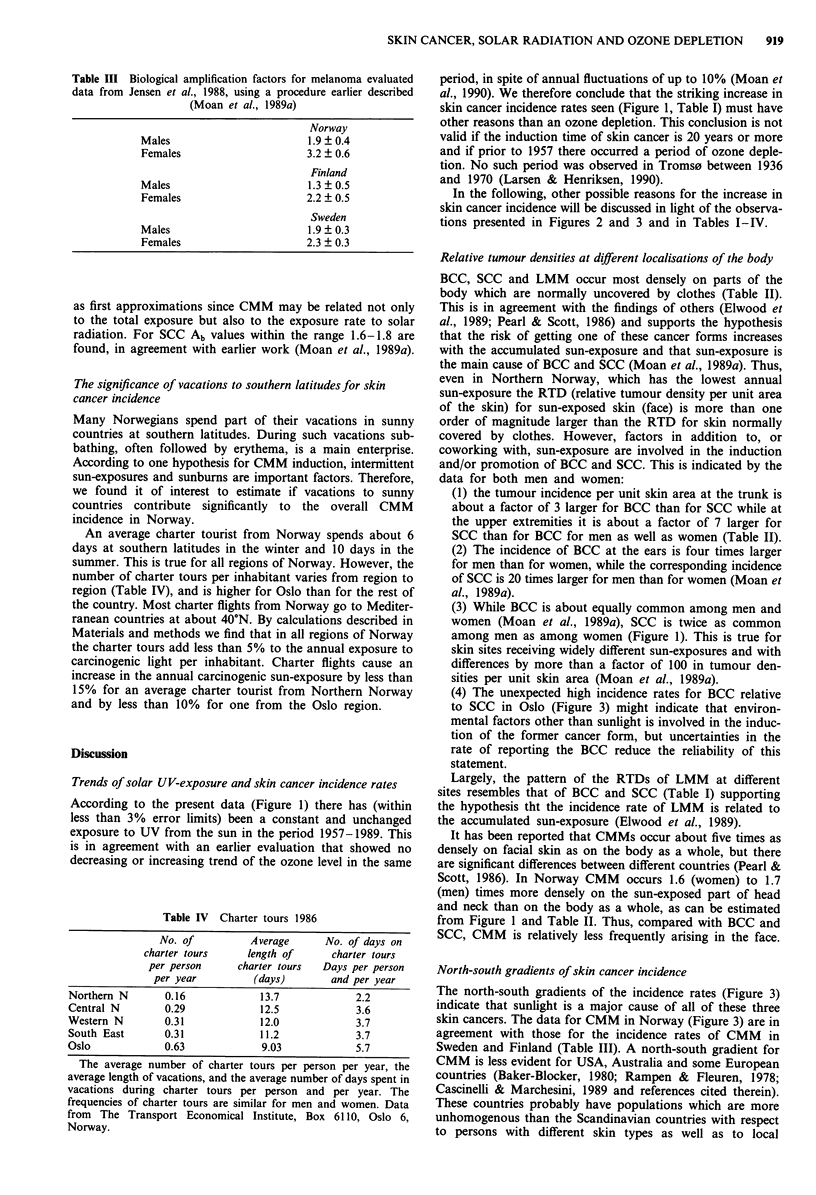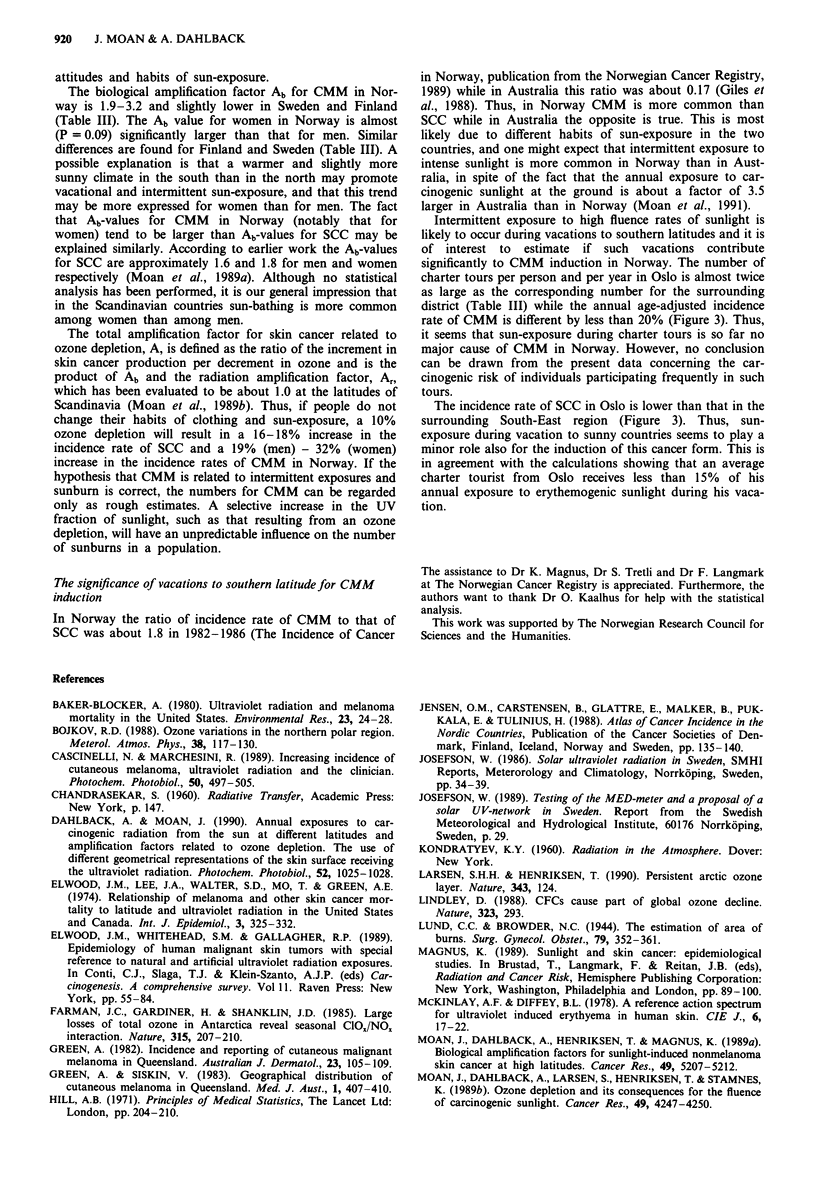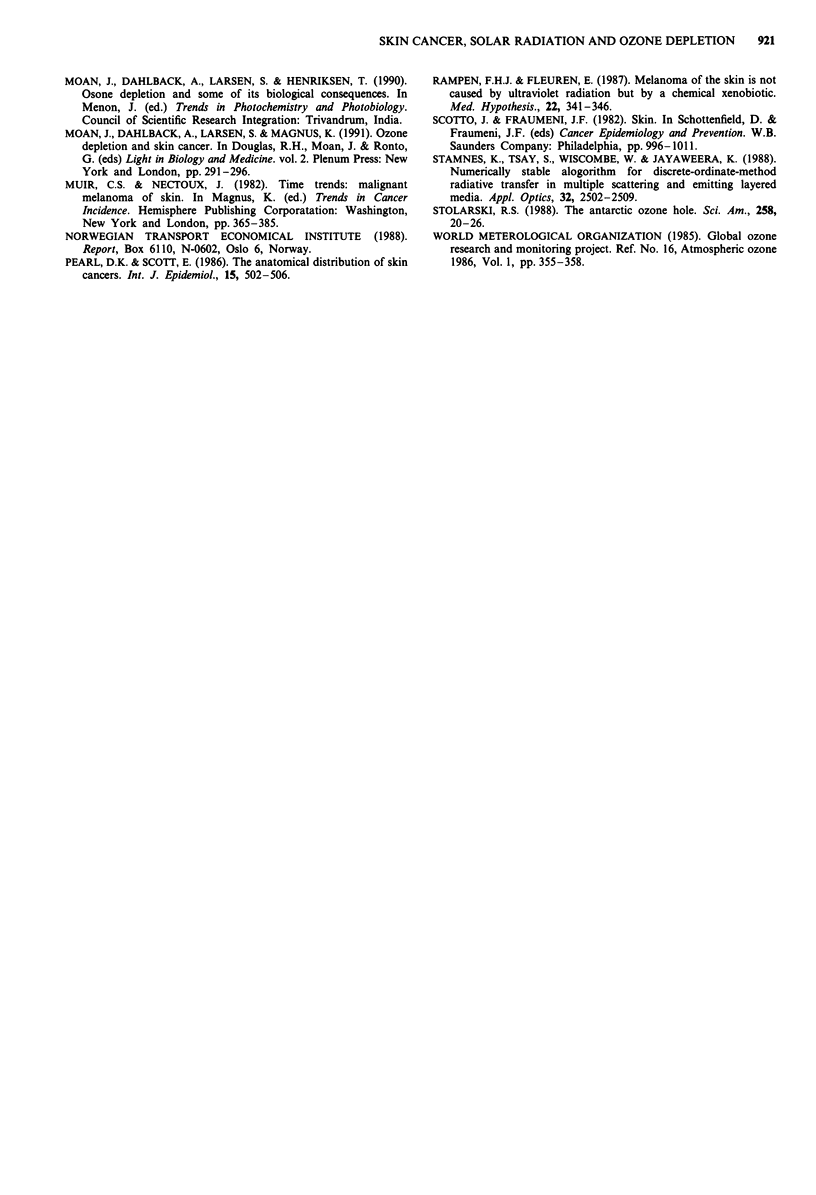# The relationship between skin cancers, solar radiation and ozone depletion.

**DOI:** 10.1038/bjc.1992.192

**Published:** 1992-06

**Authors:** J. Moan, A. Dahlback

**Affiliations:** Institute for Cancer Research, Montebello, Oslo, Norway.

## Abstract

During the period 1957-1984 the annual age-adjusted incidence rate of cutaneous malignant melanoma (CMM) increased by 350% for men and 440% for women in Norway. The annual exposure to carcinogenic sunlight in Norway, calculated by use of measured ozone levels, showed no increasing trend during the same period. Thus, ozone depletion is not a cause of the increasing trend of the incidence rates of skin cancers. The incidence rates of basal cell carcinoma (BCC) and squamous cell carcinoma (SCC) increase with decreasing latitude in Norway. The same is true for CMM in Norway, Sweden, and Finland. Our data were used to estimate the implications of a future ozone depletion for the incidence rates of skin cancer: a 10% ozone depletion was found to give rise to a 16-18% increase in the incidence rate of SCC (men and women), a 19% increase in the incidence rate of CMM for men and a 32% increase in the incidence rate of CMM for women. The difference between the numbers for men and women is almost significant and may be related to a different intermittent exposure pattern to sunlight of the two sexes. The increasing trend in the incidence rates of CMM is strongest for the trunk and lower extremities of women, followed by that for the trunk of men. The increasing incidence rates of skin cancers as well as the changing pattern of incidence on different parts of the body is most likely due to changing habits of sun exposure. Comparisons of relative densities of CMM, SCC, LMM and SCC falling per unit area of skin at different parts of the body indicate that sun exposure is the main cause of these cancer forms although other unknown factors may play significant roles as well. For the population as a whole sun exposure during vacations to sunny countries has so far been of minor importance in skin cancer induction.


Br. J. Cancer (1992), 65, 916–921 © Macmillan Press Ltd., 1992

### The relationship between skin cancers, solar radiation and ozone depletion

J. Moan[1] & A. Dahlback[2]

[1]*Institute for Cancer Research, Montebello, 0310 Oslo 3;* [2]*Norwegian Institute for Air Research, Box 64, 2001 Lilleström, Norway*

**Summary** During the period 1957–1984 the annual age-adjusted incidence rate of cutaneous malignant melanoma (CMM) increased by 350% for men and 440% for women in Norway. The annual exposure to carcinogenic sunlight in Norway, calculated by use of measured ozone levels, showed no increasing trend during the same period. Thus, ozone depletion is not a cause of the increasing trend of the incidence rates of skin cancers.

The incidence rates of basal cell carcinoma (BCC) and squamous cell carcinoma (SCC) increase with decreasing latitude in Norway. The same is true for CMM in Norway, Sweden and Finland. Our data were used to estimate the implications of a future ozone depletion for the incidence rates of skin cancer: a 10% ozone depletion was found to give rise to a 16–18% increase in the incidencee rate of SCC (men and women), a 19% increase in the incidence rate of CMM for men and a 32% increase in the incidence rate of CMM for women. The difference between the numbers for men and women is almost significant and may be related to a different intermittent exposure pattern to sunlight of the two sexes.

The increasing trend in the incidence rates of CMM is strongest for the trunk and lower extremities of women, followed by that for the trunk of men. The increasing incidence rates of skin cancers as well as the changing pattern of incidence on different parts of the body is most likely due to to changing habits of sun exposure. Comparisons of relative densities of CMM, SCC, LMM and SCC falling per unit area of skin at different parts of the body indicate that sun exposure is the main cause of these cancer forms although other unknown factors may play significant roles as well.

For the population as a whole sun exposure during vacations to sunny countries has so far been of minor importance in skin cancer induction.

There is an almost general agreement that exposure to UV-radiation from the sun is the major cause of non-melanoma skin cancers (Elwood *et al.*, 1989 and references cited therein). This is supported by north-south gradients in the incidence rates as well as by the magnitude of RTDs on body locations exposed to different doses of UV-radiation. RTD is here as the annual age adjusted rate of tumour incidence on a given site of the body divided by the proportion of the total skin surface area occupied by this site. In the southern part of Norway the incidence rates of BCC and SCC are 2.5–3 times higher than in the northern part of the country, located at about 10 degrees higher latitudes (Moan *et al.*, 1989*a*). Even in Northern Norway, where the population normally receives 40% less carcinogenic sunlight than in Southern Norway, the RTD for skin sites frequently exposed to the sun (face, head and neck) is about 30 (SCC) to 60 (BCC) times higher than that on sites normally covered by clothes (Moan *et al.*, 1989*a*).

With some exceptions (Baker-Blocker, 1980; Rampen & Fleuren, 1987; Cascinelli & Marchesini, 1989) most investigators conclude that sun exposure is a major cause also of CMM (see references in Elwood *et al.*, 1989). CMM occurs more densely on facial skin than on the body as a whole: in Australia about four times more densely (Pearl & Scott, 1986). In the Scandinavian countries as well as in USA there is a north/south gradient also for melanoma incidence (Jensen *et al.*, 1989; Scotto & Fraumeni, 1982). A north-south gradient for the mortality rates of CMM in USA has also been reported (Elwood *et al.*, 1974) although in other investigations no such gradient is found (Baker-Blocker 1980).

The incidence rates of all major forms of skin cancer have increased rapidly in most countries with white populations (Magnus, 1989; Muir & Nectoux, 1982). In Norway the incidence rate of CMM[3] doubles in 10–12 years, which is a faster rate of increase than that for any other cancer form (Magnus, 1989). In Queensland, Australia the rate of CMM increased from 16.4 per 100,000 inhibitants in 1965 to 39.6 per 100,000 in 1979–1980 (Green, 1982; Green & Siskin, 1983).

Since the Antarctic ozone hole was recognised (Farman *et al.*, 1985; Stolarski, 1988) and the Ozone Trend Panel claimed a negative trend for the ozone level of the Northern Hemisphere in the period 1969–1986 (Lindley, 1988) people have speculated if there might be an increase of the fluence of UV radiation from the sun due to the ozone depeletion and if the increasing trend in incidence rates of skin cancers might be related to such an increase.

In the present work we have applied data for annual UV-exposures calculated from known ozone values (Bojkov, 1988; Larsen & Henriksen, 1990) and epidemiological data for the period 1957–1988, to study the relationship between UV-exposure, ozone depletion and incidence rates for different types of skin cancer.

### Materials and methods

### *Carcinogenic radiation from the sun*

The fluence rate of carcinogenically effective solar radiation is defined by the expression $E_c(t) = \int E(\lambda,t)\varphi_c(\lambda)d\lambda$, the integration being performed over the wavelength region of the solar spectrum. $E(\lambda,t)$ is the solar irradiance at earth's surface, $\varphi_c(\lambda)$ is the action spectrum for carcinogenesis, and t is time.

$E(\lambda,t)$ was determined by using a discrete ordinate algorithm to calculate the propagation of light in vertically inhomogenous, plane parallel media (Stamnes *et al.*, 1988). The model atmosphere used was the US Standard Atmosphere 1976 which was divided in 39 homogenous layers with a thickness of 2 km. We used the extraterrestrial solar radiation spectra as well as all orders of scattered light (Rayleigh scattering) from the atmosphere. The ground albedo (i.e., the ratio of the upward light flux to the downward light flux (Chandrasekar, 1966)) – was set equal to 0.2 which is close to the climatological mean value for continental vegetation (Kondratyev, 1969). The absorption spectrum of ozone was

Correspondence: J. Moan.
The abbreviations used are: BCC, basal cell carcinoma; SCC, squamous cell carcinoma; LMM, lentigo maligna melanoma; CMM, cutaneous malignant melanoma; RTD, relative tumour density; UV ultraviolet.
Received 8 May 1991; and in revised form 14 January 1992.

taken from the publication 'Atmospheric ozone 1985' from World Meteorological Organization. The terrestrial spectra computed in this way agree well (both with respect to shape and absolute values) with the spectra recorded in the same area and at the same zenith angle and ozone concentration (Josefson, 1986). $\varphi_c$ for human carcinogenesis is not known. In the present work we have used the 'reference spectrum' for erythema in humans proposed by the CIE (McKinlay & Diffey, 1978). We have earlier shown that using the action spectrum for mutation of cells corrected for the transmission through the epidermis yields practically the same results (Moan *et al.*, 1989*a*).

The annual exposure to carcinogenic radiation from the sun is $D = \int E_c(t)dt$, the integral being taken over 1 year. In our calculations the integrals were approximated by the sum.

$$D = \sum\sum E(\lambda t)(\varphi)_c \Delta\lambda \Delta t$$

with $\Delta t = 1$ h and $\Delta\lambda = 1$ nm. The seasonal average ozone levels at different latitudes were used.

In all calculations the geometrical shape of the human body was approxiamated by a cylinder with its axis oriented vertically, excluding its top and bottom (Dahlback & Moan, 1990).

Mean summer and winter values for the ozone level from measurements at 12 stations north of 59°N for the period 1957–1986 (Bojkov, 1988) were used in the calculations. For the period 1987–1989 the ozone data were obtained from Larsen and Henriksen, 1990. Exposures to carcinogenic UV-light at different regions in Norway were corrected for different sky covers as outlined in (Moan *et al.*, 1989*a*).

Values for the annual UV-exposures in Norrköping, Sweden, measured by means of a Robertson Berger sunburn meter, were obtained from Dr W. Josefsson (1989).

It should be noted that throughout the present work 'exposure' means relative exposure of a cylinder oriented with its axes vertically. Possible differences in exposure habits of people in different geographical areas have not been taken into account since no relevant data exist to introduce corrections.

### *Epidemiological data*

Data for incidence rates of skin cancers were provided by the Norwegian Cancer Registry. All incidence rates were age adjusted to the European standard population (Hill, 1971).

Norway was divided in five regions: North (mean latitude 69.5°), Central (mean latitude 64°), West (mean latitude 61°), South/East (mean latitude 59.5°) and Oslo (latitude 60°). Oslo was treated separately since people living in Oslo spend significantly more time on vacations in Mediterranian countries than people in the other regions (see below). All other cities in Norway are less densely populated than Oslo and none of them are dominating in population size in their regions.

All cases of SCC, CMM and LMM are reported by the pathological laboratories to the Norwegian Cancer Registry where coding and classification takes place. Further details about the registration procedures can be found elsewhere (Jensen *et al.*, 1988).

Since not all BCC lesions are treated in hospitals, there may have been underreporting of this cancer form. To our knowledge there is no regional differences in the reporting rate of BCC although this cannot be ruled out.

Practically all inhabitants in Norway are Caucasian, and we have no reason to believe that there is any difference between different regions with respect to the distribution of persons with different skin types. When calculating the relative tumour density, RTD, on head and neck the area normally covered by hair was excluded. In the present work we use the following definition of relative tumour density:

$$RTD_s = \frac{\text{annual age adjusted incidence rate at body site s.}}{\text{fraction f of the skin surface occupied by s.}}$$

### Results

### *Trends of solar UV exposure and skin cancer incidence rates*

Figure 1 shows the annual exposure to erythemogenic radiation from the sun as a function of time for the period 1957–1989. Measurements of exposures to carcinogenic sunlight in Scandinavia exist only for Norrköping for the period 1983–1989 (Josefsson, 1989). These measurements show a larger variation from year to year than the values calculated on basis of the measured ozone levels (Figure 1, top), but have been carried out over too short a period to allow any trend analysis, The variation of the measured values is mainly due to the variation of the number of sunny days during the summer. Overall, there is no significant increasing or decreasing trend of the exposure to carcinogenic sunlight in the period analysed, and the annual fluctuations amount to only a few per cent of the mean level (Figure 1).

During the same period as the annual UV-exposure has been constant the annual age adjusted incidence rates of skin cancer have increased dramatically: by about 350% for CMM in men, by about 450% for CMM in women and by about 70% for SCC in men and women (Figure 1).

The rate of increase of the incidence of CMM (LMM excluded) is different for different sites on the body (Figure 2, Table I). For men the doubling time for incidence on head and neck is more than twice as long as the doubling time for incidence on the trunk. The doubling time for the lower extremities is also significantly longer than that for the trunk ($P < 0.008$) in the case of men, while the doubling times on these two sites are similar in the case of women (i.e. 9.6 years in both cases). A comparison of the data for men and women reveals differences: On the lower extremities the doubling time is significantly longer ($P < 0.02$) for men than for

**Figure 1** **a**, Annual exposure to carcinogenic sunlight in Norway, calculated on the basis of measured ozone values for the period 1957–1989 (—O—) and annual exposures measured in Norrköping, Sweden for the period 1983–1989 (— × —). **b**, Annual age adjusted incidence rates of SCC in men (M) and women (F) and of CMM in men (M) and women (F) for the period 1957–1984 in Norway. The points given are average values for 3 years. Representative standard errors are, as given in percentage of the values shown for 1972: 4% (CMM, F); 4% (CMM, M); 6% (SCC, F) and 4% (SCC, M).

**Figure 2** Relative values for the tumour densities (RTD) at given body sites (i.e. age-adjusted incidence rates at a given site divided by the fraction, f, of the total skin area occupied by that site) as functions of the time.

**Table I** The average time for doubling the age adjusted incidence rate of CMM at different sites during the period 1957–1987

| | *Doubling times (years)* | |
|---|---|---|
| | *Males* | *Females* |
| Whole body | 12.3 ± 0.5 | 11.2 ± 0.5 |
| Head, neck | 22.2 ± 0.6 | 16.7 ± 1.7 |
| Trunk | 8.9 ± 0.6 | 9.6 ± 0.5 |
| Lower extremities | 13.4 ± 1.2 | 9.6 ± 0.6 |

Regression analysis of data like those shown in Figure 1. Doubling times are given with standard errors.

women. On head and neck the doubling time is almost significantly longer ($P = 0.08$) for men than for women.

*Skin cancer incidence at different localisation of the body*

To evaluate the significance of solar radiation as an inducing factor of different forms of skin cancer we calculated the percentage of the total number of incidences (1976–85) that occurred at given skin sites divided by the fraction of the total skin area of the body occupied by these sites (Table II). The numbers arrived at in this way are proportional to the number of cancer incidences per unit area of skin at the given sites, i.e. to the RTD values.

**Table II** The pattern of localisation of BCC, SCC, LMM and CMM on the body

| | | *Males (%:f)* | | | |
|---|---|---|---|---|---|
| | *f* | *BCC* | *SCC* | *LMM* | *CMM*[a] |
| *Males (90:f)* | | | | | |
| Head | 0.089 | 691 | 806 | 994 | 171 |
| Trunk | 0.26 | 106 | 31 | 63 | 206 |
| Upper extremities | 0.19 | 8.0 | 59 | 17 | 53 |
| Lower extremities | 0.40 | 5.2 | 9 | 11 | 26 |
| *Females* | | | | | |
| Head | 0.089 | 720 | 784 | 774 | 161 |
| Trunk | 0.26 | 97 | 38 | 25 | 103 |
| Upper extremities | 0.19 | 7.9 | 54 | 35 | 88 |
| Lower extremities | 0.40 | 7.8 | 15 | 30 | 77 |

[a]LMM excluded, [b]All abbrevations are explained in the list of abbreviations. The numbers given are the percentage of the total number of incidences that occur at a given skin site divided by the fraction, f, of the total skin area of the body occupied by this site. Data for 1976–85. (The number for the whole body is 100 in all cases). Values are f are from Lund and Browder, 1988.

*Skin cancer incidence rates at geographical localisations with different levels of annual exposure to carcinogenic sunlight*

The incidence rates of BCC, SCC and CMM increase with increasing annual exposure to carcinogenic sunlight (Figure 3). Data for BCC and SCC have been published earlier (Moan *et al.*, 1989*a*) but are included for comparison.

Data, such as those shown in Figure 3, can be used to evaluate the impact of an ozone depletion on the incidence rates of skin cancer by use of the action spectrum and the numerical calculations earlier described (Moan *et al.*, 1989*a*). The biological amplification factor, $A_b$, is defined as the ratio of the increment in skin cancer production to the increment in causative sunlight exposure. According to the present data for CMM in Norway the value of $A_b$ is 1.9 for men and 3.2 for women (Table III). These values should be regarded only

**Figure 3** The age-adjusted incidence rates of BCC, SCC and CMM (excluding LMM) at different regions of Norway expressed as percentages of the mean values for the country and as functions of the annual exposure to carcinogenic sunlight.

**Table III** Biological amplification factors for melanoma evaluated data from Jensen *et al.*, 1988, using a procedure earlier described (Moan *et al.*, 1989*a*)

| | |
|---|---|
| | *Norway* |
| Males | 1.9 ± 0.4 |
| Females | 3.2 ± 0.6 |
| | *Finland* |
| Males | 1.3 ± 0.5 |
| Females | 2.2 ± 0.5 |
| | *Sweden* |
| Males | 1.9 ± 0.3 |
| Females | 2.3 ± 0.3 |

as first approximations since CMM may be related not only to the total exposure but also to the exposure rate to solar radiation. For SCC $A_b$ values within the range 1.6–1.8 are found, in agreement with earlier work (Moan *et al.*, 1989*a*).

*The significance of vacations to southern latitudes for skin cancer incidence*

Many Norwegians spend part of their vacations in sunny countries at southern latitudes. During such vacations sub-bathing, often followed by erythema, is a main enterprise. According to one hypothesis for CMM induction, intermittent sun-exposures and sunburns are important factors. Therefore, we found it of interest to estimate if vacations to sunny countries contribute significantly to the overall CMM incidence in Norway.

An average charter tourist from Norway spends about 6 days at southern latitudes in the winter and 10 days in the summer. This is true for all regions of Norway. However, the number of charter tours per inhabitant varies from region to region (Table IV), and is higher for Oslo than for the rest of the country. Most charter flights from Norway go to Mediterranean countries at about 40°N. By calculations described in Materials and methods we find that in all regions of Norway the charter tours add less than 5% to the annual exposure to carcinogenic light per inhabitant. Charter flights cause an increase in the annual carcinogenic sun-exposure by less than 15% for an average charter tourist from Northern Norway and by less than 10% for one from the Oslo region.

### Discussion

*Trends of solar UV-exposure and skin cancer incidence rates*

According to the present data (Figure 1) there has (within less than 3% error limits) been a constant and unchanged exposure to UV from the sun in the period 1957–1989. This is in agreement with an earlier evaluation that showed no decreasing or increasing trend of the ozone level in the same

**Table IV** Charter tours 1986

| | *No. of charter tours per person per year* | *Average length of charter tours (days)* | *No. of days on charter tours Days per person and per year* |
|---|---|---|---|
| Northern N | 0.16 | 13.7 | 2.2 |
| Central N | 0.29 | 12.5 | 3.6 |
| Western N | 0.31 | 12.0 | 3.7 |
| South East | 0.31 | 11.2 | 3.7 |
| Oslo | 0.63 | 9.03 | 5.7 |

The average number of charter tours per person per year, the average length of vacations, and the average number of days spent in vacations during charter tours per person and per year. The frequencies of charter tours are similar for men and women. Data from The Transport Economical Institute, Box 6110, Oslo 6, Norway.

period, in spite of annual fluctuations of up to 10% (Moan *et al.*, 1990). We therefore conclude that the striking increase in skin cancer incidence rates seen (Figure 1, Table I) must have other reasons than an ozone depletion. This conclusion is not valid if the induction time of skin cancer is 20 years or more and if prior to 1957 there occurred a period of ozone depletion. No such period was observed in Tromsø between 1936 and 1970 (Larsen & Henriksen, 1990).

In the following, other possible reasons for the increase in skin cancer incidence will be discussed in light of the observations presented in Figures 2 and 3 and in Tables I–IV.

*Relative tumour densities at different localisations of the body*

BCC, SCC and LMM occur most densely on parts of the body which are normally uncovered by clothes (Table II). This is in agreement with the findings of others (Elwood *et al.*, 1989; Pearl & Scott, 1986) and supports the hypothesis that the risk of getting one of these cancer forms increases with the accumulated sun-exposure and that sun-exposure is the main cause of BCC and SCC (Moan *et al.*, 1989*a*). Thus, even in Northern Norway, which has the lowest annual sun-exposure the RTD (relative tumour density per unit area of the skin) for sun-exposed skin (face) is more than one order of magnitude larger than the RTD for skin normally covered by clothes. However, factors in addition to, or coworking with, sun-exposure are involved in the induction and/or promotion of BCC and SCC. This is indicated by the data for both men and women:

(1) the tumour incidence per unit skin area at the trunk is about a factor of 3 larger for BCC than for SCC while at the upper extremities it is about a factor of 7 larger for SCC than for BCC for men as well as women (Table II).

(2) The incidence of BCC at the ears is four times larger for men than for women, while the corresponding incidence of SCC is 20 times larger for men than for women (Moan *et al.*, 1989*a*).

(3) While BCC is about equally common among men and women (Moan *et al.*, 1989*a*), SCC is twice as common among men as among women (Figure 1). This is true for skin sites receiving widely different sun-exposures and with differences by more than a factor of 100 in tumour densities per unit skin area (Moan *et al.*, 1989*a*).

(4) The unexpected high incidence rates for BCC relative to SCC in Oslo (Figure 3) might indicate that environmental factors other than sunlight is involved in the induction of the former cancer form, but uncertainties in the rate of reporting the BCC reduce the reliability of this statement.

Largely, the pattern of the RTDs of LMM at different sites resembles that of BCC and SCC (Table I) supporting the hypothesis tht the incidence rate of LMM is related to the accumulated sun-exposure (Elwood *et al.*, 1989).

It has been reported that CMMs occur about five times as densely on facial skin as on the body as a whole, but there are significant differences between different countries (Pearl & Scott, 1986). In Norway CMM occurs 1.6 (women) to 1.7 (men) times more densely on the sun-exposed part of head and neck than on the body as a whole, as can be estimated from Figure 1 and Table II. Thus, compared with BCC and SCC, CMM is relatively less frequently arising in the face.

*North-south gradients of skin cancer incidence*

The north-south gradients of the incidence rates (Figure 3) indicate that sunlight is a major cause of all of these three skin cancers. The data for CMM in Norway (Figure 3) are in agreement with those for the incidence rates of CMM in Sweden and Finland (Table III). A north-south gradient for CMM is less evident for USA, Australia and some European countries (Baker-Blocker, 1980; Rampen & Fleuren, 1978; Cascinelli & Marchesini, 1989 and references cited therein). These countries probably have populations which are more unhomogenous than the Scandinavian countries with respect to persons with different skin types as well as to local attitudes and habits of sun-exposure.

The biological amplification factor $A_b$ for CMM in Norway is 1.9–3.2 and slightly lower in Sweden and Finland (Table III). The $A_b$ value for women in Norway is almost ($P = 0.09$) significantly larger than that for men. Similar differences are found for Finland and Sweden (Table III). A possible explanation is that a warmer and slightly more sunny climate in the south than in the north may promote vacational and intermittent sun-exposure, and that this trend may be more expressed for women than for men. The fact that $A_b$-values for CMM in Norway (notably that for women) tend to be larger than $A_b$-values for SCC may be explained similarly. According to earlier work the $A_b$-values for SCC are approximately 1.6 and 1.8 for men and women respectively (Moan *et al.*, 1989*a*). Although no statistical analysis has been performed, it is our general impression that in the Scandinavian countries sun-bathing is more common among women than among men.

The total amplification factor for skin cancer related to ozone depletion, A, is defined as the ratio of the increment in skin cancer production per decrement in ozone and is the product of $A_b$ and the radiation amplification factor, $A_r$, which has been evaluated to be about 1.0 at the latitudes of Scandinavia (Moan *et al.*, 1989*b*). Thus, if people do not change their habits of clothing and sun-exposure, a 10% ozone depletion will result in a 16–18% increase in the incidence rate of SCC and a 19% (men) – 32% (women) increase in the incidence rates of CMM in Norway. If the hypothesis that CMM is related to intermittent exposures and sunburn is correct, the numbers for CMM can be regarded only as rough estimates. A selective increase in the UV fraction of sunlight, such as that resulting from an ozone depletion, will have an unpredictable influence on the number of sunburns in a population.

*The significance of vacations to southern latitude for CMM induction*

In Norway the ratio of incidence rate of CMM to that of SCC was about 1.8 in 1982–1986 (The Incidence of Cancer in Norway, publication from the Norwegian Cancer Registry, 1989) while in Australia this ratio was about 0.17 (Giles *et al.*, 1988). Thus, in Norway CMM is more common than SCC while in Australia the opposite is true. This is most likely due to different habits of sun-exposure in the two countries, and one might expect that intermittent exposure to intense sunlight is more common in Norway than in Australia, in spite of the fact that the annual exposure to carcinogenic sunlight at the ground is about a factor of 3.5 larger in Australia than in Norway (Moan *et al.*, 1991).

Intermittent exposure to high fluence rates of sunlight is likely to occur during vacations to southern latitudes and it is of interest to estimate if such vacations contribute significantly to CMM induction in Norway. The number of charter tours per person and per year in Oslo is almost twice as large as the corresponding number for the surrounding district (Table III) while the annual age-adjusted incidence rate of CMM is different by less than 20% (Figure 3). Thus, it seems that sun-exposure during charter tours is so far no major cause of CMM in Norway. However, no conclusion can be drawn from the present data concerning the carcinogenic risk of individuals participating frequently in such tours.

The incidence rate of SCC in Oslo is lower than that in the surrounding South-East region (Figure 3). Thus, sun-exposure during vacation to sunny countries seems to play a minor role also for the induction of this cancer form. This is in agreement with the calculations showing that an average charter tourist from Oslo receives less than 15% of his annual exposure to erythemogenic sunlight during his vacation.

The assistance to Dr K. Magnus, Dr S. Tretli and Dr F. Langmark at The Norwegian Cancer Registry is appreciated. Furthermore, the authors want to thank Dr O. Kaalhus for help with the statistical analysis.

This work was supported by The Norwegian Research Council for Sciences and the Humanities.

### References

BAKER-BLOCKER, A. (1980). Ultraviolet radiation and melanoma mortality in the United States. *Environmental Res.*, **23**, 24–28.

BOJKOV, R.D. (1988). Ozone variations in the northern polar region. *Meterol. Atmos. Phys.*, **38**, 117–130.

CASCINELLI, N. & MARCHESINI, R. (1989). Increasing incidence of cutaneous melanoma, ultraviolet radiation and the clinician. *Photochem. Photobiol.*, **50**, 497–505.

CHANDRASEKAR, S. (1960). *Radiative Transfer*, Academic Press: New York, p. 147.

DAHLBACK, A. & MOAN, J. (1990). Annual exposures to carcinogenic radiation from the sun at different latitudes and amplification factors related to ozone depletion. The use of different geometrical representations of the skin surface receiving the ultraviolet radiation. *Photochem. Photobiol.*, **52**, 1025–1028.

ELWOOD, J.M., LEE, J.A., WALTER, S.D., MO, T. & GREEN, A.E. (1974). Relationship of melanoma and other skin cancer mortality to latitude and ultraviolet radiation in the United States and Canada. *Int. J. Epidemiol.*, **3**, 325–332.

ELWOOD, J.M., WHITEHEAD, S.M. & GALLAGHER, R.P. (1989). Epidemiology of human malignant skin tumors with special reference to natural and artificial ultraviolet radiation exposures. In Conti, C.J., Slaga, T.J. & Klein-Szanto, A.J.P. (eds) *Carcinogenesis. A comprehensive survey.* Vol 11. Raven Press: New York, pp. 55–84.

FARMAN, J.C., GARDINER, H. & SHANKLIN, J.D. (1985). Large losses of total ozone in Antarctica reveal seasonal $ClO_x/NO_x$ interaction. *Nature*, **315**, 207–210.

GREEN, A. (1982). Incidence and reporting of cutaneous malignant melanoma in Queensland. *Australian J. Dermatol.*, **23**, 105–109.

GREEN, A. & SISKIN, V. (1983). Geographical distribution of cutaneous melanoma in Queensland. *Med. J. Aust.*, **1**, 407–410.

HILL, A.B. (1971). *Principles of Medical Statistics*, The Lancet Ltd: London, pp. 204–210.

JENSEN, O.M., CARSTENSEN, B., GLATTRE, E., MALKER, B., PUKKALA, E. & TULINIUS, H. (1988). *Atlas of Cancer Incidence in the Nordic Countries*, Publication of the Cancer Societies of Denmark, Finland, Iceland, Norway and Sweden, pp. 135–140.

JOSEFSON, W. (1986). *Solar ultraviolet radiation in Sweden*, SMHI Reports, Meterorology and Climatology, Norrköping, Sweden, pp. 34–39.

JOSEFSON, W. (1989). *Testing of the MED-meter and a proposal of a solar UV-network in Sweden.* Report from the Swedish Meteorological and Hydrological Institute, 60176 Norrköping, Sweden, p. 29.

KONDRATYEV, K.Y. (1960). *Radiation in the Atmosphere.* Dover: New York.

LARSEN, S.H.H. & HENRIKSEN, T. (1990). Persistent arctic ozone layer. *Nature*, **343**, 124.

LINDLEY, D. (1988). CFCs cause part of global ozone decline. *Nature*, **323**, 293.

LUND, C.C. & BROWDER, N.C. (1944). The estimation of area of burns. *Surg. Gynecol. Obstet.*, **79**, 352–361.

MAGNUS, K. (1989). Sunlight and skin cancer: epidemiological studies. In Brustad, T., Langmark, F. & Reitan, J.B. (eds), *Radiation and Cancer Risk*, Hemisphere Publishing Corporation: New York, Washington, Philadelphia and London, pp. 89–100.

MCKINLAY, A.F. & DIFFEY, B.L. (1978). A reference action spectrum for ultraviolet induced erythyema in human skin. *CIE J.*, **6**, 17–22.

MOAN, J., DAHLBACK, A., HENRIKSEN, T. & MAGNUS, K. (1989*a*). Biological amplification factors for sunlight-induced nonmelanoma skin cancer at high latitudes. *Cancer Res.*, **49**, 5207–5212.

MOAN, J., DAHLBACK, A., LARSEN, S., HENRIKSEN, T. & STAMNES, K. (1989*b*). Ozone depletion and its consequences for the fluence of carcinogenic sunlight. *Cancer Res.*, **49**, 4247–4250.

MOAN, J., DAHLBACK, A., LARSEN, S. & HENRIKSEN, T. (1990). Osone depletion and some of its biological consequences. In Menon, J. (ed.) *Trends in Photochemistry and Photobiology*. Council of Scientific Research Integration: Trivandrum, India.
MOAN, J., DAHLBACK, A., LARSEN, S. & MAGNUS, K. (1991). Ozone depletion and skin cancer. In Douglas, R.H., Moan, J. & Ronto, G. (eds) *Light in Biology and Medicine*. vol. 2. Plenum Press: New York and London, pp. 291–296.
MUIR, C.S. & NECTOUX, J. (1982). Time trends: malignant melanoma of skin. In Magnus, K. (ed.) *Trends in Cancer Incidence*. Hemisphere Publishing Corporatation: Washington, New York and London, pp. 365–385.
NORWEGIAN TRANSPORT ECONOMICAL INSTITUTE (1988). *Report*, Box 6110, N-0602, Oslo 6, Norway.
PEARL, D.K. & SCOTT, E. (1986). The anatomical distribution of skin cancers. *Int. J. Epidemiol.*, **15**, 502–506.
RAMPEN, F.H.J. & FLEUREN, E. (1987). Melanoma of the skin is not caused by ultraviolet radiation but by a chemical xenobiotic. *Med. Hypothesis.*, **22**, 341–346.
SCOTTO, J. & FRAUMENI, J.F. (1982). Skin. In Schottenfield, D. & Fraumeni, J.F. (eds) *Cancer Epidemiology and Prevention*. W.B. Saunders Company: Philadelphia, pp. 996–1011.
STAMNES, K., TSAY, S., WISCOMBE, W. & JAYAWEERA, K. (1988). Numerically stable alogorithm for discrete-ordinate-method radiative transfer in multiple scattering and emitting layered media. *Appl. Optics*, **32**, 2502–2509.
STOLARSKI, R.S. (1988). The antarctic ozone hole. *Sci. Am.*, **258**, 20–26.
WORLD METEROLOGICAL ORGANIZATION (1985). Global ozone research and monitoring project. Ref. No. 16, Atmospheric ozone 1986, Vol. 1, pp. 355–358.